# A Nitronaphthalimide Probe for Fluorescence Imaging of Hypoxia in Cancer Cells

**DOI:** 10.1007/s10895-021-02800-6

**Published:** 2021-08-12

**Authors:** Rashmi Kumari, Vasumathy R, Dhanya Sunil, Raghumani Singh Ningthoujam, Badri Narain Pandey, Suresh D. Kulkarni, Thivaharan Varadavenkatesan, Ganesh Venkatachalam, Anil Kumar N. V

**Affiliations:** 1 Department of Chemistry, Manipal Institute of Technology, Manipal Academy of Higher Education, Manipal, 576104 Karnataka India; 2 Radiation Biology & Health Sciences Division, Bhabha Atomic Research Centre, Mumbai, 400085 Maharashtra India; 3 Chemistry Division, Bhabha Atomic Research Centre, Mumbai, 400085 Maharashtra India; 4 Department of Atomic and Molecular Physics, Manipal Academy of Higher Education, Manipal, 576104 Karnataka India; 5 Department of Biotechnology, Manipal Institute of Technology, Manipal Academy of Higher Education, Manipal, 576104 Karnataka India; 6 Electrodics and Electrocatalysis (EEC) Division, CSIR – Central Electrochemical Research Institute (CSIR-CECRI), Karaikudi, 630003 Tamil Nadu India; 7 Homi Bhabha National Institute, Anushakti Nagar, Mumbai, 400094 India

**Keywords:** Docking, Fluorescence, Hypoxia, Molecular probe, Nitronaphthalimide, Nitroreductase

## Abstract

The bioreductive enzymes typically upregulated in hypoxic tumor cells can be targeted for developing diagnostic and drug delivery applications. In this study, a new fluorescent probe 4−(6−nitro−1,3−dioxo−1*H*−benzo[*de*]isoquinolin−2(3*H*)−yl)benzaldehyde (NIB) based on a nitronaphthalimide skeleton that could respond to nitroreductase (NTR) overexpressed in hypoxic tumors is designed and its application in imaging tumor hypoxia is demonstrated. The docking studies revealed favourable interactions of NIB with the binding pocket of NTR-*Escherichia coli*. NIB, which is synthesized through a simple and single step imidation of 4−nitro−1,8−naphthalic anhydride displayed excellent reducible capacity under hypoxic conditions as evidenced from cyclic voltammetry investigations. The fluorescence measurements confirmed the formation of identical products (NIB-red) during chemical as well as NTR−aided enzymatic reduction in the presence of NADH. The potential fluorescence imaging of hypoxia based on NTR-mediated reduction of NIB is confirmed using *in-vitro* cell culture experiments using human breast cancer (MCF−7) cells, which displayed a significant change in the fluorescence colour and intensity at low NIB concentration within a short incubation period in hypoxic conditions.

**Graphical abstract:**

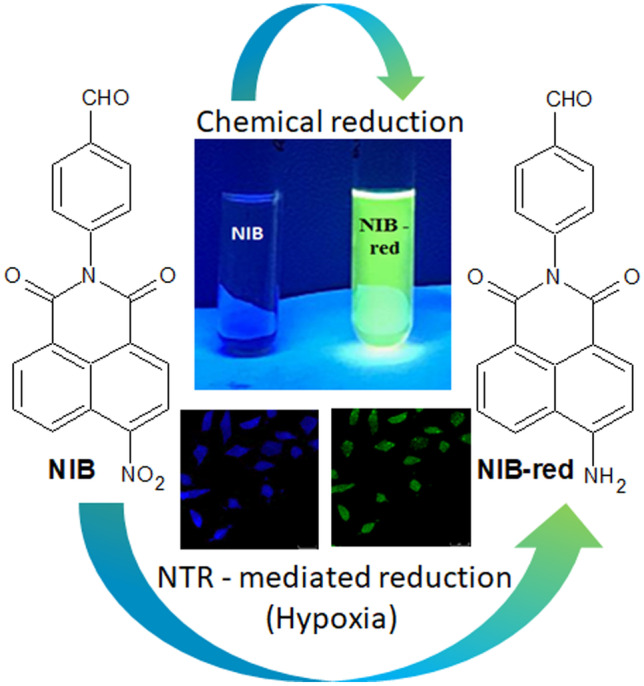

**Supplementary Information:**

The online version contains supplementary material available at 10.1007/s10895-021-02800-6.

## Introduction

Cancer is a pathological condition wherein cells undergo abnormal and uncontrolled division in an alarming rate to form a malignant tumor which can subsequently invade the surrounding tissues and later metastasize to secondary locations [1]. As per World Health Organization (WHO), this dreadful disease occupies second leading position next to cardiac diseases in its fatality rate [2]. The highly proliferating tumors with altered metabolism demand more supply of nutrition and oxygen that trigger the abrupt formation of chaotic and defective microvessels. These impaired, but adaptive vasculature networks limit the adequate supply of oxygen into the cells located deep within the growing tumor mass, as the diffusion of oxygen is possible only to ~ 200 μm [3, 4]. Therefore, hypoxia is a distinctive pathological condition recognized in most of the solid tumors, wherein the inner cores of the tumor do not receive sufficient supply of oxygen [5–7]. These malignant cells develop hypoxia-related resistance to various cancer therapies, and can result in altered dose–response profiles for cancer therapeutics in clinical scenario [8, 9]. As normal cellular environment does not comprise of these unique and hostile settings perceived in hypoxic tumours, the oxygen gradient in solid tumors can be exploited for developing hypoxia based cancer imaging to aid diagnostic purposes, and also in fabricating hypoxia specific drug delivery systems for cancer therapy. 

The heterogenic hypoxic microenvironment in advancing tumors are very often characterized by lower pH that results from increased anaerobic respiration, and thereby leads to a bioreductive environment due to upregulation of reductase enzymes [10, 11]. There are reports on nitroaromatic, azobenzene or quinone derivatives that are successfully employed as molecular probes for imaging cellular oxygen levels and as hypoxia-sensitive pro-drugs, which are activated through various reductases that accumulate within the tumor due to oxygen stress [12–16]. Among the various types of hypoxia responsive molecules investigated as imaging probes and pro-drugs till date, hydrophobic nitronaphthalimides are a class of well-recognized substrates for nitroreductase (NTR) enzyme expressed abundantly in anaerobic environments [17–22]. These nitro derivatives occupy a superior position because of its ability to undergo a series of stepwise bioreductive processes to form nitroso (2e^−^), hydroxylamine (4e^−^) and finally highly fluorescent aminonaphthalimides (6e^−^) in the presence of reduced β-nicotinamide adenine dinucleotide (NADH) as the electron donor [23–25]. With this rationale, a new chemical entity 4−(6−nitro−1,3−dioxo−1*H*−benzo[*de*]isoquinolin-2(3*H*)−yl)benzaldehyde **(**NIB) which integrates a benzaldehyde unit into 4−nitro−1,8−nitronaphthalimide chemical skeleton was designed and prepared through an easy one step imidation of 4−nitro−1,8−naphthalic anhydride using 4−amino benzaldehyde. The nitro moiety which is a typical fluorescence quencher can act as a suitable substrate for NTR and can transform into a highly fluorescent amino derivative in hypoxic medium with the prospective of an imaging probe. Moreover, the aldehyde functional group can enable its conjugation with drug delivery carriers for fabrication of a potential hypoxia responsive drug vehicle. Besides, the site-specific delivery of the payloads in hypoxic cancers can also be monitored using fluorescence measurements. The characteristic optical features of NIB after exposure to chemical, electrochemical, enzymatic, and in vitro biological reductive environments were investigated. The probe NIB with an intrinsic weak blue emission exhibited a hypoxia-triggered intense green fluorescence and could be a potentially useful candidate as a diagnostic tool for fluorescence imaging of hypoxia or could possibly be conjugated with drug delivery vehicles to develop hypoxia sensitive theranostics.

## Experimental

### Reagents

The precursor materials (analytical grade) and the solvents used for the synthesis were purchased from Sigma and Spectrochem respectively, and were used without further purification. NTR from *Escherichia coli* (*E. coli*−N9284) and reduced β- Nicotinamide adenine dinucleotide disodium salt hydrate (NADH−HPLC grade N8129) for enzyme related experiments were procured from Sigma. Phosphate-buffered saline (PBS: pH = 7.4) solution was purchased from Himedia. The lyophilized NTR powder was dissolved in PBS for use and the solution was frozen instantly at -20 °C for subsequent storage. Ultrapure water (over 18 MΩ·cm) from a Milli-Q reference system (Elix-Merck Millipore) was used for all the experiments.

### In-silico Docking Studies

#### Rationale for Choosing NTR with PDB ID: 3X21

There are no human NTR crystal structures available in protein data bank (PDB). Moreover, a survey of the PDB site revealed only two crystal structures of *E. coli* with resolutions of 2.0 Å and 3.0 Å, reported since 2015. Hence, the crystal structure of *E. coli* NTR that belongs to oxidoreductase category with higher resolution of 3.0 Å (PDB ID: 3X21) and R-Value of 0.256 was selected as the protein target for docking studies with NIB as ligand [26].

### Protein Preparation

The interaction of NIB with NTR was investigated through molecular docking using Schrodinger Suite 11.8 (Maestro -11.8, Sitemap) software. X-ray crystal structure of NTR was downloaded from RCSB protein data bank. The protein was prepared using the Protein Preparation Wizard of Schrodinger Suite 11.8 (Schrodinger, Inc.) and preprocessed. Bond orders were assigned, hydrogens were added, metals were treated, water molecules were deleted and heterostate for co-crystallized ligand was generated using Epic, protonation state. The hydrogen bonding optimization of the protein side chain was assigned using Protasign. Energy was minimized (impref minimization) by means of OPLS 3e force field. The ligand structure was sketched using 2D sketcher of Maestro-11.8 and prepared employing the Ligprep module of Schrodinger Suite using default parameters set at pH 7 ± 2 using OPLS 3e force field. Receptor grid was prepared without constrains and generated using receptor grid generation panel around the vicinity of co-crystalline ligand. Default values were accepted for van der Waals scaling and partial input charges were used. The glide tool (maestro 11.8) was used to dock prepared ligand against the generated receptor grid of protein. The default parameters for scaling factor and partial charge cutoff site was specified as centroid of the selected residues. The final docking study was carried out using extra precision docking method, and all possible self and cross docking studies were performed using the extra precision scoring function.

### Synthesis and Characterization

4-(6-nitro-1,3-dioxo-1*H*-benzo[*de*]isoquinolin-2(3*H*)-yl)benzaldehyde (NIB): 4-Aminobenzaldehyde (0.605 g, 5 mmol) was added to 6-nitro-1*H*,3*H*-naphtho[1,8-*cd*]pyran-1,3-dione (1.216 g, 5 mmol) dissolved in ethanol (20 mL), and refluxed for 43 h at 80 ºC. The reaction mixture was cooled down to room temperature, poured into ice cold water to get a red precipitate of NIB, which was filtered, dried and recrystallized from ethanol.

**Scheme 1.**
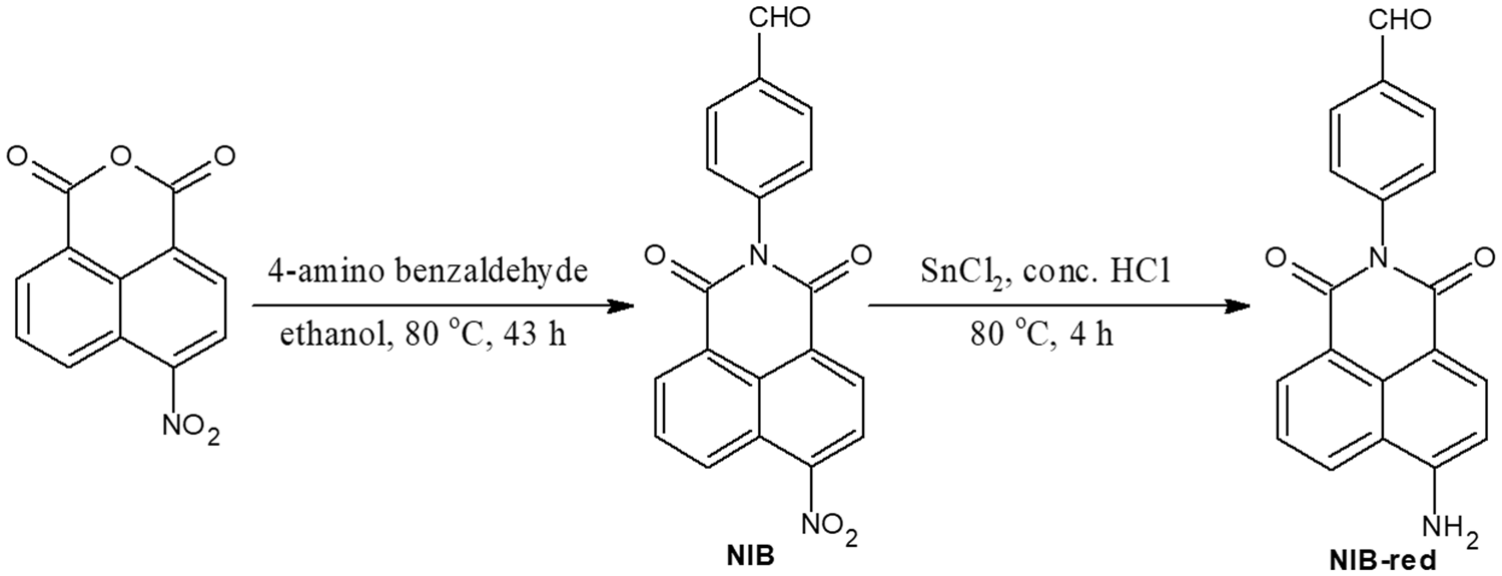
Synthetic route for NIB and NIB-red

4-(6-amino-1,3-dioxo-1*H*-benzo[*de*]isoquinolin-2(3*H*)-yl)benzaldehyde (NIB-red): Tin (II) chloride (0.568 g, 3 mmol) in 2 mL of concentrated hydrochloric acid was added dropwise to NIB (0.173 g, 0.5 mmol) dissolved in 2 mL of concentrated hydrochloric acid, and heated at 80 °C for 4 h. The precipitate was filtered, washed with water, dried and recrystallized to obtain NIB-red. The synthetic pathway for NIB and NIB-red is presented in Scheme 1.

The reaction progress was monitored by thin layer chromatography using pre-coated aluminium sheet. Melting points were measured by open capillary method and are uncorrected. The IR spectra in KBr pellets and NMR (^1^H and ^13^C) spectra using TMS as internal standard and DMSO-*d6*/CDCl_3_ as solvent were recorded using Shimadzu FTIR spectrophotometer and 400 MHz Bruker spectrometer, respectively. The electronic spectra were taken using 1800 Shimadzu UV–visible spectrophotometer, and JASCO spectrofluorometer FP 8300 was employed to record the emission spectra of the naphthalimide derivatives.

### Cyclic Voltammetry (CV) Measurements

The reduction potential of 1 mmol NIB was examined in an electrochemical analyser (BioLogic Model: SP-240 procured from France) with Ag/Ag^+^, glassy carbon and Pt wire as the reference, working and auxiliary electrodes respectively, immersed in 0.1 M tetrabutylammonium tetrafluoroborate (TBAFB_4_) in DMSO, with Ferrocene as an internal reference. The measurements were taken in DMSO, DMSO + 1 mmol NIB and DMSO + 1 mmol NIB + 0.5 mM NADH initially under normal atmospheric conditions and later with nitrogen saturated solutions. The potential was varied from -1.5 to + 1.5 V at a scan rate of 150 mV/s [27].

### Enzymatic Reduction Studies

About 3.46 mg of NIB was dissolved in 50 mL DMSO to prepare 200 µM stock solution. Further, stock solutions of 200 µg/mL NTR and 1000 µM NADH were prepared in PBS. The experiments were carried out by withdrawing required quantities from the respective stock solutions and further diluting using PBS. The time-dependent fluorescence measurements were recorded by maintaining the concentration of NIB, NTR and NADH as 1 μM, 20 μg/mL and 500 μM respectively. Kinetic studies were carried out by varying the NTR concentration in presence of 500 μM NADH [13, 28].

### Cell Culture

MCF-7 (human breast cancer) cells were procured from European Collection of Authenticated Cell Cultures (ECACC), Sigma Aldrich. These cells were cultured in Dulbecco’s modified Eagle’s medium (Gibco) supplemented with 10% fetal calf serum (FCS; Himedia, Mumbai) and antibiotics (100 U/mL Penicillin and 100 μg/mL Streptomycin; Sigma) at 37 ºC, 20% O_2_ in a 5% CO_2_ atmosphere (also used for normoxia cultures). Cells were maintained in an exponentially growing culture condition and passaged twice a week. Unless specified, cells were harvested by trypsinisation followed by washing with PBS. For achieving hypoxic conditions, the cells were incubated in a hypoxic chamber with cell imaging system (H800, Bioxia India, Mumbai, India) at 1% O_2_, 5% CO_2_, 37 ºC and ~ 95% humidity.

### Staining of Cells with NIB and Fluorescence Imaging

MCF-7 cells (1 × 10^6^) were seeded in 30 mm Petri plates. Before treatment, the cells were cultured under normoxic conditions for 24 h. Then the cells were treated with 6 µM NIB in culture medium. The cells to be incubated under hypoxic conditions were shifted to hypoxic chamber with cell imaging system (Lumascope 620, Etaluma Inc., CA, USA), and the cells to be incubated under normoxic conditions were left in the CO_2_ incubator under routine culture conditions. After different time points (2, 4, 6, 8 and 24 h), cells incubated under hypoxic or normoxic conditions were imaged for bright and blue/green fluorescence (blue: λ_exc_ = 370–410 nm and λ_em_ = 429–462 nm and green: λ_exc_ = 473–491 nm and λ_em_ = 502–561 nm). At 6 h, cells were also imaged after washing with PBS to remove any dye leaked from the cells. The raw intensities of the images were quantified using Image J (NIH, USA), an open-source image processing software. The ratio of fluorescent intensities in blue to green images of the cells were calculated for hypoxic and normoxic conditions, and further these ratios were normalised with respect to normoxic ratios to calculate percentage conversion of fluorescent intensities from blue to green. Relative fluorescent intensities of blue to green at different time points of hypoxic cells were normalised with respect to corresponding normoxic cells.

## Results and Discussion

### In silico Molecular Modelling Studies

**Fig. 1:**
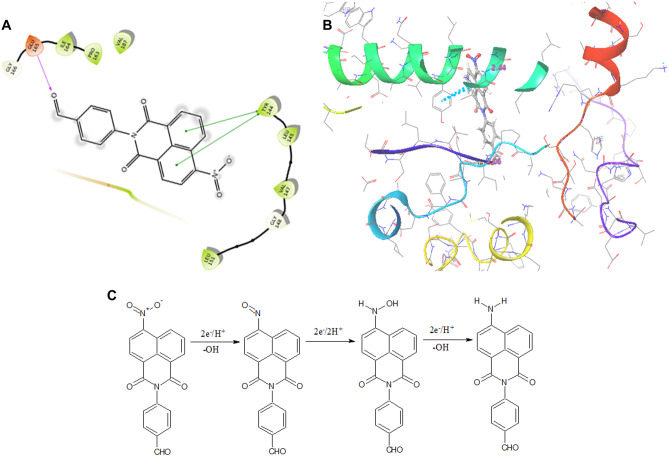
(**A**) 2D view of molecular interactions of NIB with amino acid residues of NTR: hydrogen bond interactions are shown in purple arrow, the amino acid residues that form hydrophobic contacts are presented in green and π- π stacking with green arrows. (**B**) Overlay of NIB in the active binding pocket of 3X21. (**C**) Proposed reduction of NIB in presence of NTR to NIB-red

The malignant cells which are under oxidative stress in growing tumors typically show increased expression of NTR [29]. Hence, to investigate the affinity of the designed substrate NIB towards NTR, in silico molecular docking studies were performed. The molecular interactions of NIB with amino acid residues of NTR are presented in Fig. 1. NIB exhibited hydrophobic interactions with ILE 164, PRO 163, VAL 147, VAL 187, TYR 144, LEU 145, and LEU 151 amino acid residues located within the binding pocket of NTR. A hydrogen bonding association between the oxygen of the aldehyde functional group and hydrogen of N–H of GLU 165 residue (2.06 Å) was observed. Moreover, NIB also engaged in π-π stacking interaction with TYR 144 residue. The ligand NIB displayed a docking score of -2.841. These facilitated interactions can enable NIB to be a good substrate for NTR and its subsequent biological reduction to form the NIB-red product in hypoxic environment [28] as portrayed in Fig. 1.

### Characterization of NIB

Based on the good docking score and the interactions between the substrate NIB and the enzyme NTR, the nitronaphthalimide derivative NIB was synthesized. The spectral data of NIB was in agreement with its chemical structure.

4-(6-nitro-1,3-dioxo-1*H*-benzo[*de*]isoquinolin-2(3*H*)-yl)benzaldehyde (NIB) Reddish brown; 63%; m.p. 195–198 ºC; UV–Vis (λ_abs_, nm): 346 (Fig. S1); PL (λ_em_, nm): 433 (Fig. S2A); IR (cm^−1^): 1357, 1525 (−NO_2_ *str.*), 1587 (C = C *str.*), 1672, 1714, 1784 (C = O *str.*), 2862, 2924 (C-H *str.*), 3080 (Ar. C-H *str.*) (Fig. S3); ^1^H NMR (400 MHz, DMSO-*d6*): 7.259–7.280 (d, 2H, 8.4 Hz), 7.321–7.342 (d, 2H, 8.4 Hz), 8.101–8.141 (d, 1H, 8.8 Hz), 8.565–8.600 (m, 2H, 7.6 Hz), 8.620–8.645 (d, 1H, 7.6 Hz), 8.741–8.765 (d, 1H, 8.8 Hz), 10.1 (CHO) (Fig. S4); ^13^C NMR (100 MHz, DMSO-*d6*): 127.95, 128.24, 128.40, 129.16, 129.28, 130.32, 130.66, 131.90, 132.00, 132.48, 132.51 132.70, 133.72, 136.28, 144.85, 193.28 (Fig. S5).

### Electrochemical Studies to Determine Reduction Potential

**Fig. 2:**
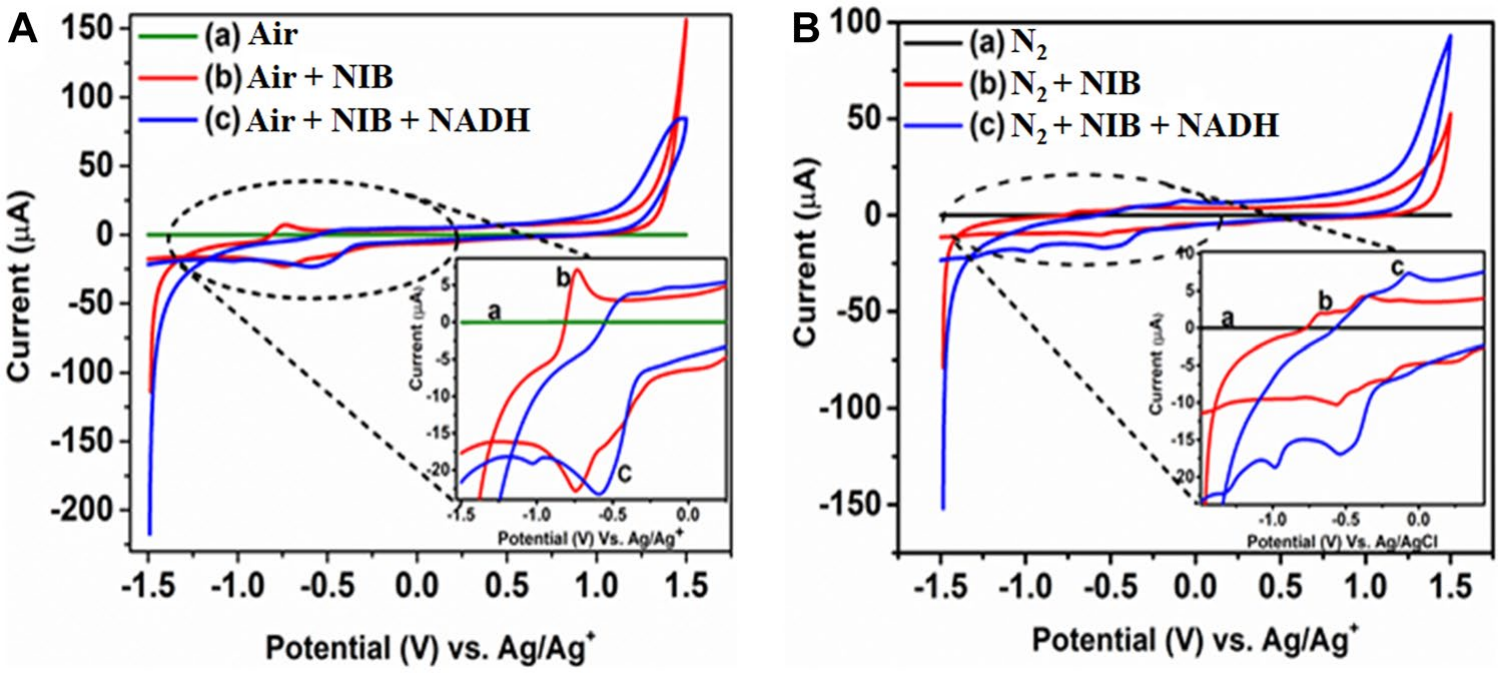
Cyclic voltammograms recorded for 1 mmol of NIB alone and NIB in presence of 0.5 mM NADH solution in 0.1 M TBAFB_4_ (DMSO) at (**A**) normal oxygen environment (normoxic) and (**B**) nitrogen environment (hypoxic)

In order to examine whether NIB can be reduced, CV experiments were performed in both normoxic and hypoxic conditions, and the recorded voltammograms are presented in Fig. 2. Interestingly, under the hypoxic environment created by purging nitrogen in the electrolyte system, the reduction potential of NIB was found to be shifted to higher values compared to normoxic environment. A similar trend was also observed when the experiment was further repeated in the presence of NADH. This indicates that the reduction of NIB is facile under hypoxia that leads to more product formation, which in turn aids in better imaging. Moreover, an enhanced reduction in hypoxia over normoxia is observed upon adding NADH in the system, when compared to NIB alone. This makes the probe more resistant to reduction in normoxic conditions. Thus the CV measurements confirmed the reduction capacity of NIB under hypoxic condition, comparable to earlier reports on nitronaphthalimides [30].

### Chemical Reduction of NIB

As NIB displayed excellent reducible ability in hypoxic medium, which is significantly higher than the normoxic environment, the chemical reduction of NIB was carried out in presence of stannous chloride and hydrochloric acid. The nitro group in the weakly blue emissive NIB was converted into amino moiety to form NIB-red, which emitted intensively to display green fluorescence. The functional group transformation in the reduced product NIB-red was confirmed through spectral characterization. The absorbance peak which was observed at 346 nm in 1 × 10^–4^ M NIB in DMSO was hypsochromically shifted to 276 nm in case of its reduction product, NIB-red due to the restricted conjugation (Fig. S1). The photoluminescence spectra of 1 × 10^–4^ M solution of NIB in DMSO exhibited a blue emission at 433 nm with low intensity attributed to the fluorescence quenching effect of NO_2_ moiety, whereas that of NIB-red displayed a ten-fold intense green fluorescence at 540 nm (Fig. S2B). The photographs depicting the variations in the fluorescence emission of NIB before and after chemical reduction under UV lamp (λ_ex_ = 365 nm) is presented in Fig. S2C. IR spectrum of the reduced product exhibited two N–H stretching bands characteristic of primary amines at 3246 and 3350 cm^−1^. Similarly, ^1^HNMR spectra of both NIB and NIB-red displayed the aromatic protons and aldehydic proton from 7.259 to 8.765 ppm and 10.106 ppm for NIB and 6.842 to 8.586 ppm and 10.040 ppm in case of NIB-red. Moreover, the ^1^HNMR spectra of NIB-red exhibited a new broad singlet at 4.919 ppm attributed to the amine protons confirming the chemical reduction of NIB.

4-(6-amino-1,3-dioxo-1*H*-benzo[*de*]isoquinolin-2(3*H*)-yl)benzaldehyde (NIB-red) Dark brown; 59%; m.p. 240-242 ºC; UV-Vis (λ_abs_, nm): 276 (Fig. S1); PL (λ_em_, nm) (Fig. S2B): 540; IR (cm^-1^): 1579 (C=C *str.*), 1643, 1683 (C=O *str.*), 2860, 2924 (C-H *str.*), 3041 (Ar. C-H *str.*) 3246, 3350 (NH_2_ *str.*) (Fig. S6); ^1^H NMR (400 MHz, CDCl_3_): 4.919 (s, 2H, NH_2_), 6.842-6.862 (d, 1H, 8 Hz), 7.110-7.128 (d, 2H, 7.2 Hz), 7.256-7.274 (d, 2H, 7.2 Hz), 7.604-7.644 (t, 1H, 8.4 Hz), 8.075-8.097 (d, 1H, 8.8 Hz), 8.383-8.403 (d, 1H, 8 Hz), 8.568-8.586 (d, 1H, 7.2 Hz), 10.040 (CHO) (Fig. S7).

### NTR-Mediated Reduction of NIB

**Fig. 3:**
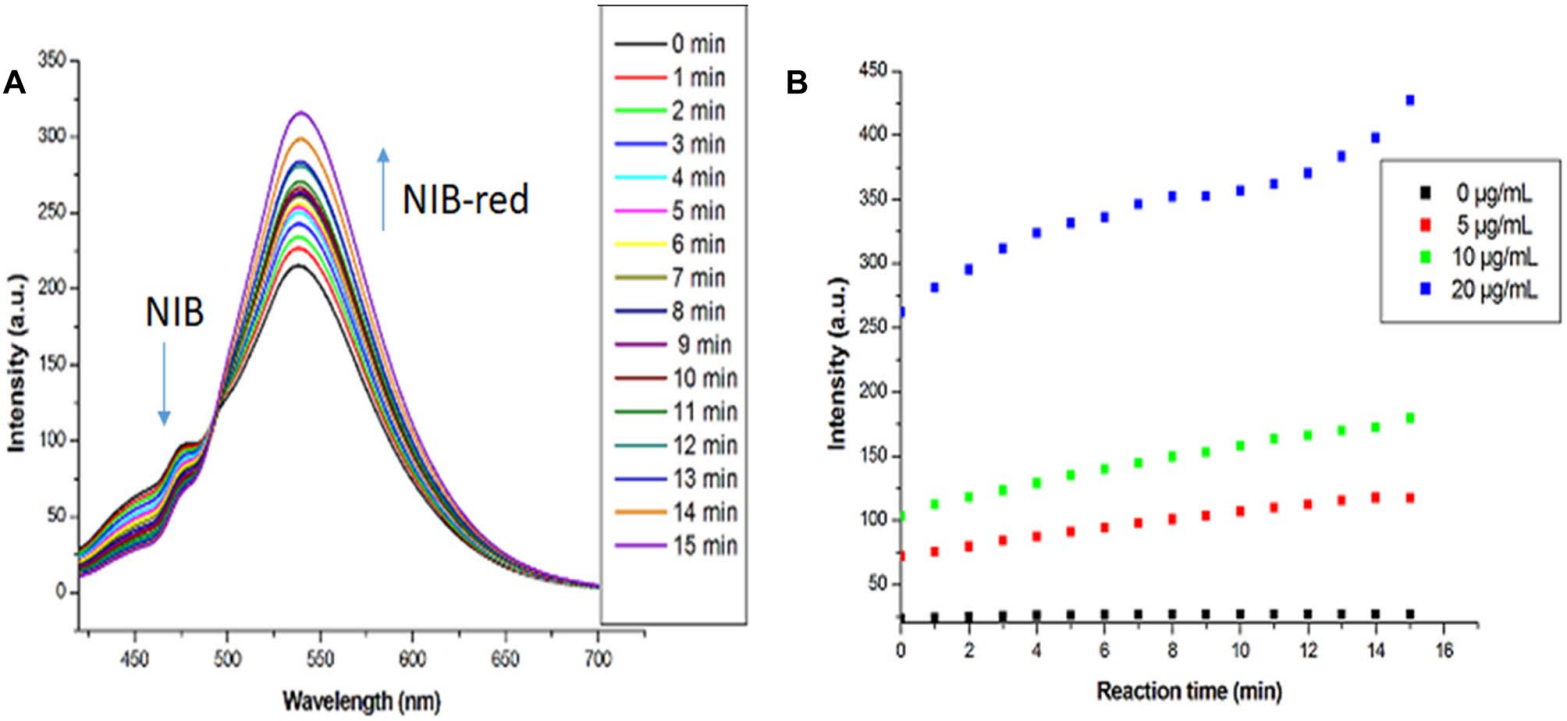
(**A**) Time-dependent variation in the fluorescence emission of 1 μM NIB in PBS (pH = 7.4) with 1% DMSO in the presence of 20 μg/mL NTR and 500 μM NADH (λ_exc_ = 410 nm) and (**B**) Fluorescence intensity of 1 μM NIB-red *vs.* the reaction time on treatment with different concentrations of NTR (0 to 20 µg/mL) in presence of 500 µM NADH (λ_exc_ = 433 nm)

The less fluorescent nitroaromatics can be reduced by NTR overexpressed in the hypoxic environment into aminoaromatics which can display enhanced emission. Based on the docking score of NIB to NTR and the intense fluorescence emission of chemically reduced NIB-red, further investigations on the fluorescence spectral features of NIB during NTR-assisted enzymatic reduction were performed in presence of NADH under physiological conditions at room temperature. All the fluorescence measurements were carried out after mixing NTR with all other constituents, and the earliest measurement having a time gap of 15 s. The time-dependent variation in the emission wavelength and intensity during the NTR mediated NIB reduction to form NIB-red is presented in Fig. 3A. The fluorescence emission maxima of NIB-red was observed at 539 nm in presence of NTR and NADH, which is comparable to that obtained in DMSO after chemical reduction. This confirms the enzymatic reduction of the nitro group in the weakly blue-emitting NIB to the amino group of the intense green fluorophore NIB-red. At the end of 15 min, though the fluorescence intensity of NIB decreased, it was not completely quenched, which may be attributed to incomplete biological reaction. Further, the kinetics of NIB (1 µM) reduction was investigated by varying the NTR concentration, and the measured fluorescence kinetic curves are shown in Fig. 3B. The study indicated a rise in fluorescence intensity with increasing NTR concentration due to a faster reduction process. In contrast, the fluorescence of NIB hardly changed with time in the absence of NTR, which implied its stability in the detection system. The chemical and enzymatic studies demonstrate the ability of NIB to perform as a successful sensor for NTR mediated bioreduction in hypoxic cells with abundant NTR concentration.

### Imaging of Human Breast Cancer Cells Under Normoxic and Hypoxic Conditions

**Fig. 4:**
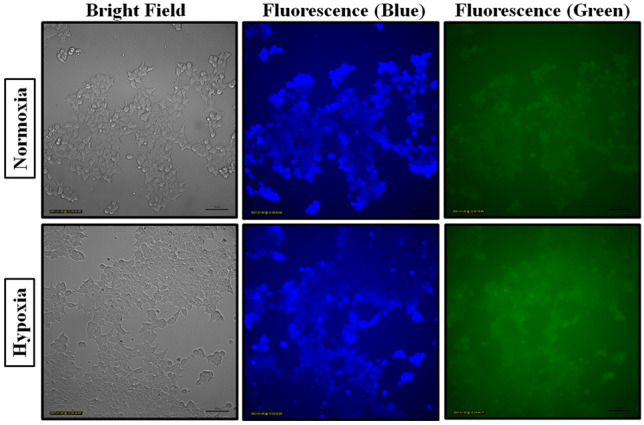
Fluorescence images of MCF-7 cells under normoxic (20% O_2_) and hypoxic (1% O_2_) conditions after 2 h of NIB incubation, imaged for bright and blue/green fluorescence (blue: λ_exc_ = 370−410 nm; λ_em_ = 429–462 nm; green: λ_exc_ = 473–491 nm; λ_em_ = 502–561 nm). Scale bar: 100 µM

**Fig. 5:**
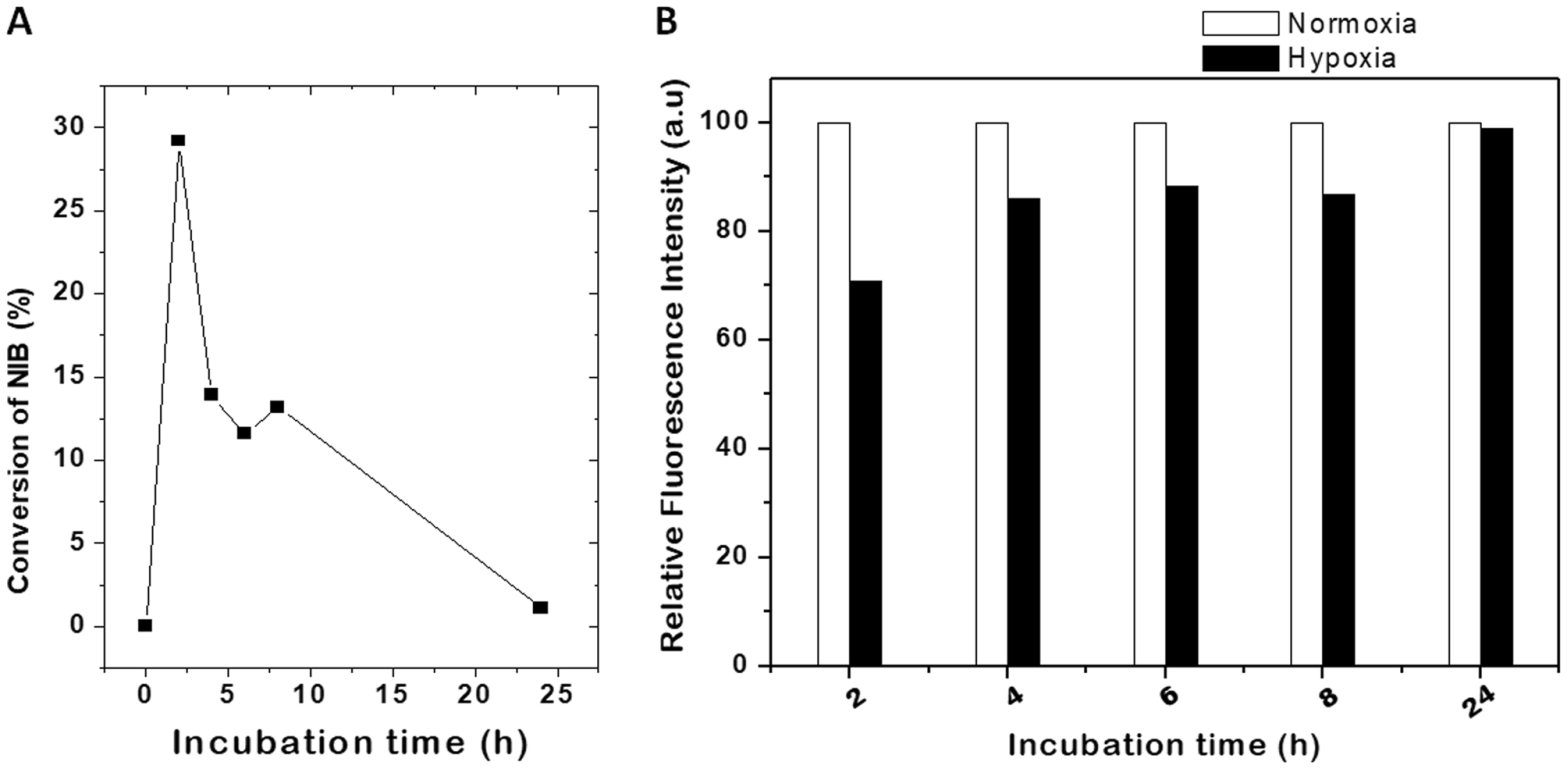
(**A**) Percentage conversion of fluorescence intensities and (**B**) relative fluorescence intensity from blue (λ_em_ = 478 nm) to green (λ_em_ = 539 nm) wavelength in hypoxic conditions with respect to control as mentioned in materials and methods. The raw intensities of the images were calculated using Image J software and percentage of conversion was calculated by normalising the intensities with respect to control. The values are average of two independent experiments

In order to study the potential of NIB for hypoxia imaging applications, MCF-7 cells were incubated with 6 µM NIB under hypoxic and normoxic conditions at 37 ºC for 0, 2, 4, 6, 8 and 24 h for fluorescence imaging experiments. The fluorescence images were acquired with suitable optical filters at blue and green regions. The blue fluorescence in the normoxic conditions in Fig. 4 indicates that the probe is cell permeable. Cells treated with NIB under hypoxia conditions showed increase in green fluorescence intensity suggesting conversion of NIB to NIB-red under hypoxia conditions. Typical images of MCF-7 cells treated with NIB under normoxia and hypoxia conditions are shown in Fig. 4. Images acquired at different incubation periods were quantified for fluorescence intensity using Image J software (NIH, USA). The values obtained for blue and green wavelengths and their ratios were used to study the kinetics of NIB conversion under normoxic and hypoxic conditions (Fig. 5). Maximum reduction of NIB was found to occur in the initial 2 h (Fig. 5A) and further decrease at longer incubation periods, which suggest saturation of NIB conversion at longer periods due to limited availability of either NIB or cellular reductase enzyme. These results were also evident in the measurement of relative fluorescence intensities under hypoxic and normoxic conditions as depicted in Fig. 5B. The relative fluorescence of NIB under hypoxic conditions at 2 h was found to be lower, and later increased with incubation periods and reached to ~ 98% at 24 h.

While doing experiments, in addition to cells, green fluorescence was also observed in the background of cell culture, which was found to increase with incubation periods (data not shown). Such background fluorescence may be due to leakage of enzymatically converted NIB under hypoxia conditions. To prove the same, for a particular incubation period i.e. 6 h, one set of culture was washed with pre-warmed fresh culture medium and used for imaging. The acquired images exhibited a decrease in the fluorescence in the background of cells after washing (Fig. S8 (before washing) and Fig. S9 (after washing)), suggesting the leakage of formed product after intracellular reduction by NTR. These results confirm the cellular internalization of NIB and conversion of NIB under hypoxic conditions. Moreover, these results may also suggest the non-toxic nature of the probe NIB as the NTR-mediated reduction was possible only because the cells could remain viable during the process.

## Conclusions

The present study demonstrated the design of a nitronaphthalimide derivative NIB which displayed excellent molecular interactions in docking studies with *E. coli*−NTR. The new probe was synthesized and investigated for its capability to act as a fluorescent marker in hypoxic environments. NIB revealed a distinct variation in colour and intensity of fluorescence in response to NTR−mediated reduction in presence of NADH. Further, the formation of amino naphthalimide NIB−red through NTR−catalyzed reaction was established through chemical reduction experiments. The cell viability and fluorescence imaging investigations demonstrated the ability of biocompatible NIB to potentially differentiate between normoxia and hypoxia through endogenous bioreductase at the cellular level. The change in fluorescence colour and intensity within a short duration of incubation of NIB under in vitro conditions could be extended to other cancer cells as well as in vivo tumor conditions for its potential in-vivo applications in tumor hypoxia imaging.

## Supplementary Information

Below is the link to the electronic supplementary material.

Supplementary file1 (DOCX 3825 KB)
