# Supplementary material for: A Nitronaphthalimide Probe for Fluorescence Imaging of Hypoxia in Cancer Cells
Source: J Fluoresc. 2021 Aug 12;31(6):1665–73. doi: 10.1007/s10895-021-02800-6 (PMC8545720; doi:10.1007/s10895-021-02800-6)
Supplement: Supplementary file 1 — Supplementary file1 (DOCX 3825 KB) [file 10895_2021_2800_MOESM1_ESM.docx]

**A Nitronaphthalimide Probe for Fluorescence Imaging of Hypoxia in Cancer Cells**

Rashmi Kumari^1^, Vasumathy R^2^, Dhanya Sunil^1^**^*^**, Raghumani Singh Ningthoujam^3,7^, Badri Narain Pandey^2,7^, Suresh D Kulkarni^4^, Thivaharan Varadavenkatesan^5^, Ganesh Venkatachalam^6^, Anil Kumar N V ^1^

^1^Department of Chemistry, Manipal Institute of Technology, Manipal Academy of Higher Education, Manipal-576104, Karnataka, India

^2^Radiation Biology & Health Sciences Division, Bhabha Atomic Research Centre, Mumbai-400085, Maharashtra, India

^3^Chemistry Division, Bhabha Atomic Research Centre, Mumbai-400085, Maharashtra, India

^4^Department of Atomic and Molecular Physics, Manipal Academy of Higher Education, Manipal-576104, Karnataka, India

^5^Department of Biotechnology, Manipal Institute of Technology_,_ Manipal Academy of Higher Education, Manipal-576104, Karnataka, India

^6^Electrodics and Electrocatalysis (EEC) Division, CSIR – Central Electrochemical Research Institute (CSIR-CECRI), Karaikudi-630003, Tamil Nadu, India

^7^Homi Bhabha National Institute, Anushakti Nagar, Mumbai-400094, India

**^*^ Corresponding author**

Dr. Dhanya Sunil,

Senior Associate Professor,

Department of Chemistry,

Manipal Institute of Technology,

Manipal, Karnataka, India.

E-mail: dhanya.s@manipal.edu

**Supplementary data**

**Supplementary figures**

| **Sl. No** | **Figure number** | **Figure caption** |
| --- | --- | --- |
| 1 | S1 | Electronic spectra of 1 × 10^-4^ M solutions of NIB and NIB-red in DMSO |
| 2 | S2 | Emission spectrum of 1 × 10^-4^ M solutions of A) NIB and B) NIB-red in DMSO. C) The fluorescence of 1 × 10^-4^ M solutions of NIB and NIB-red in DMSO observed under UV lamp (λ_exc_.: 365 nm). |
| 3 | S3 | FT-IR spectrum of NIB |
| 4 | S4 | ^1^H NMR spectrum of NIB |
| 5 | S5 | ^13^C NMR spectrum of NIB |
| 6 | S6 | FT-IR spectrum of NIB-red |
| 7 | S7 | ^1^H NMR spectrum of NIB-red |
| 8 | S8 | Fluorescence images of MCF-7 cells incubated with 6 µM NIB under normoxic (20% O_2_) and hypoxic (1% O_2_) conditions, after 6 h of NIB incubation before washing |
| 9 | S9 | Fluorescence images of MCF-7 cells incubated with 6 µM NIB under normoxic (20% O_2_) and hypoxic (1% O_2_) conditions, after 6 h of NIB incubation imaged after washing |


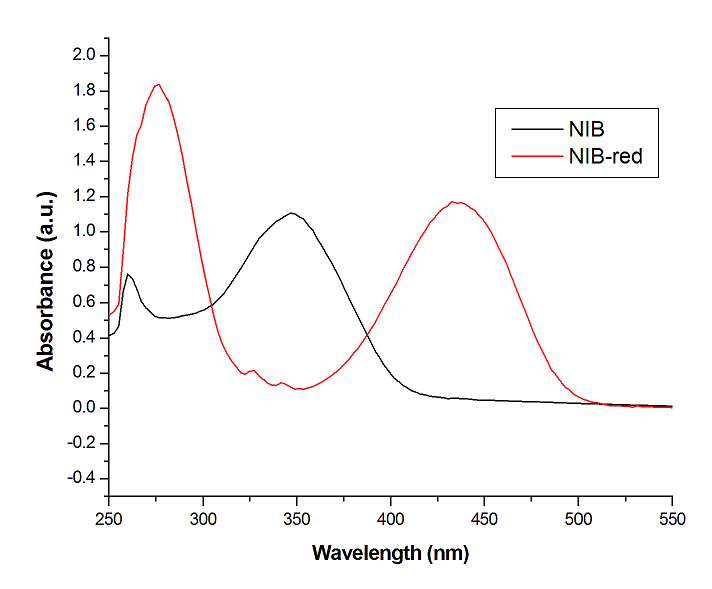


**Fig. S1** Electronic spectra of 1 × 10^-4^ M solutions of NIB and NIB-red in DMSO


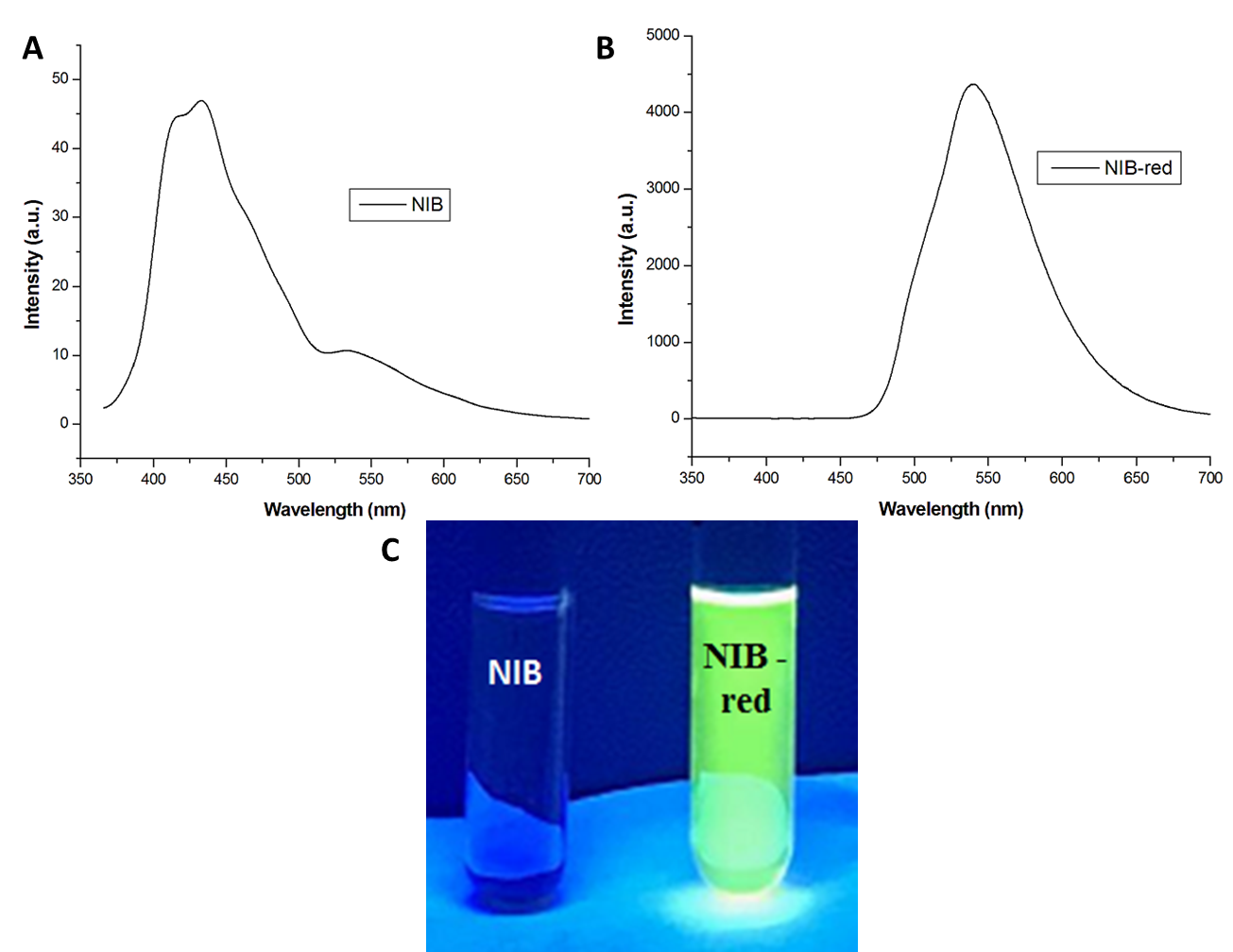


**Fig. S2** Emission spectrum of 1 × 10^-4^ M solutions of A) NIB and B) NIB-red in DMSO. C) The fluorescence of 1 × 10^-4^ M solutions of NIB and NIB-red in DMSO observed under UV lamp (λ_exc_.: 365 nm).


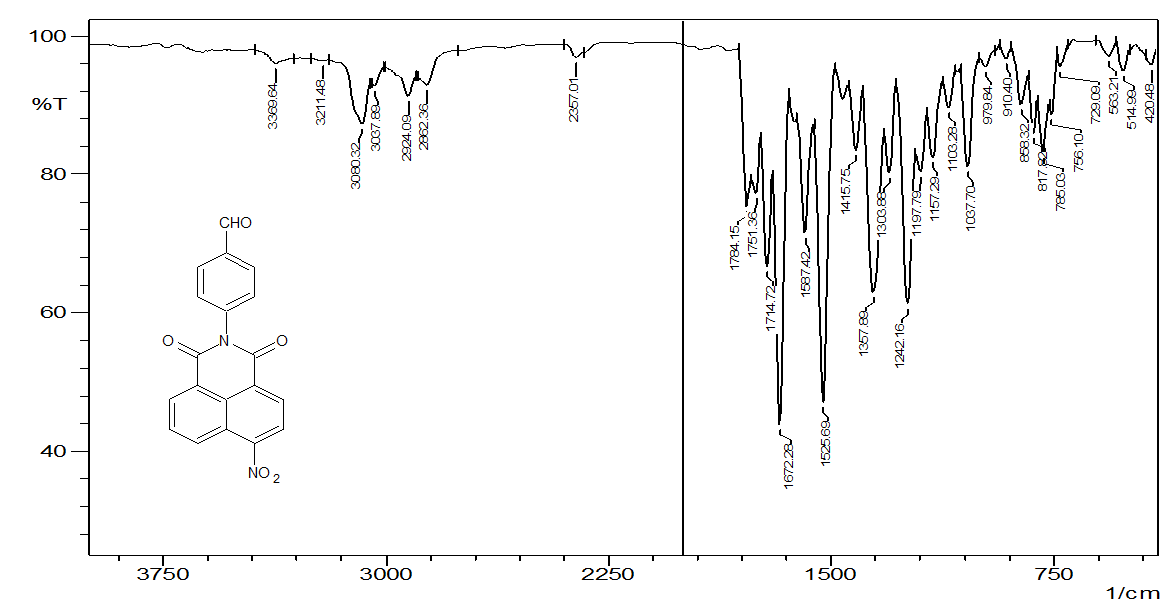


**Fig. S3** FT-IR spectrum of NIB


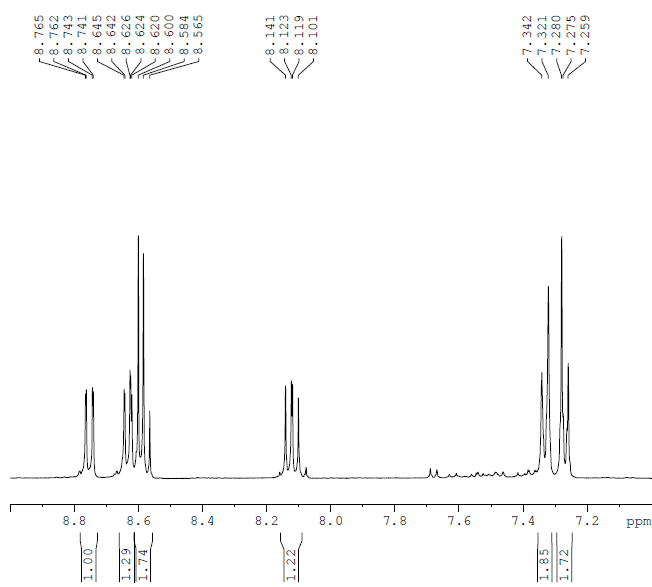

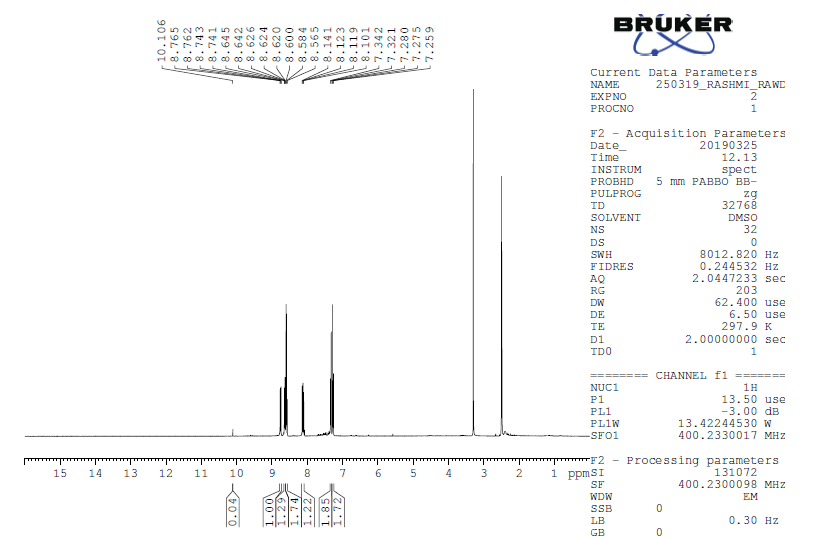


**Fig. S4** ^1^H NMR spectrum of NIB


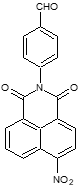

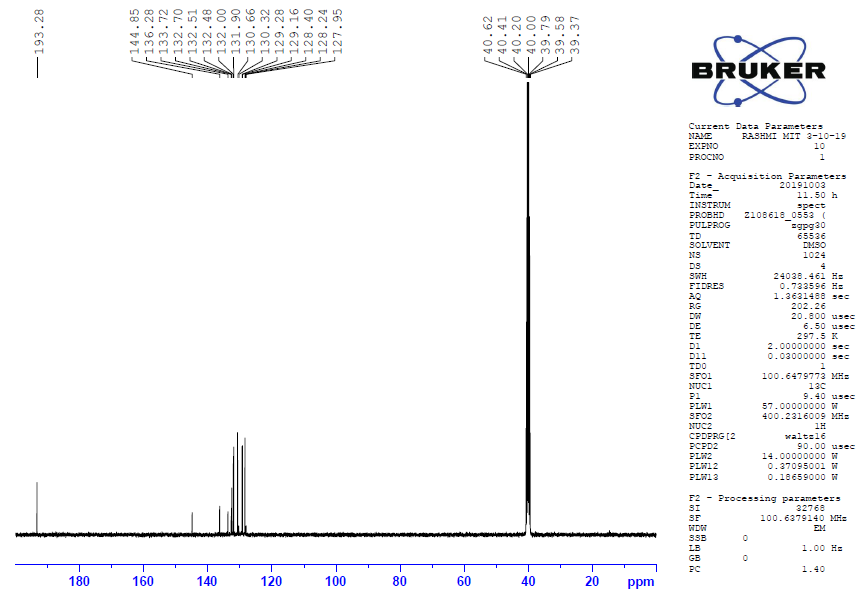


**Fig. S5** ^13^C NMR spectrum of NIB


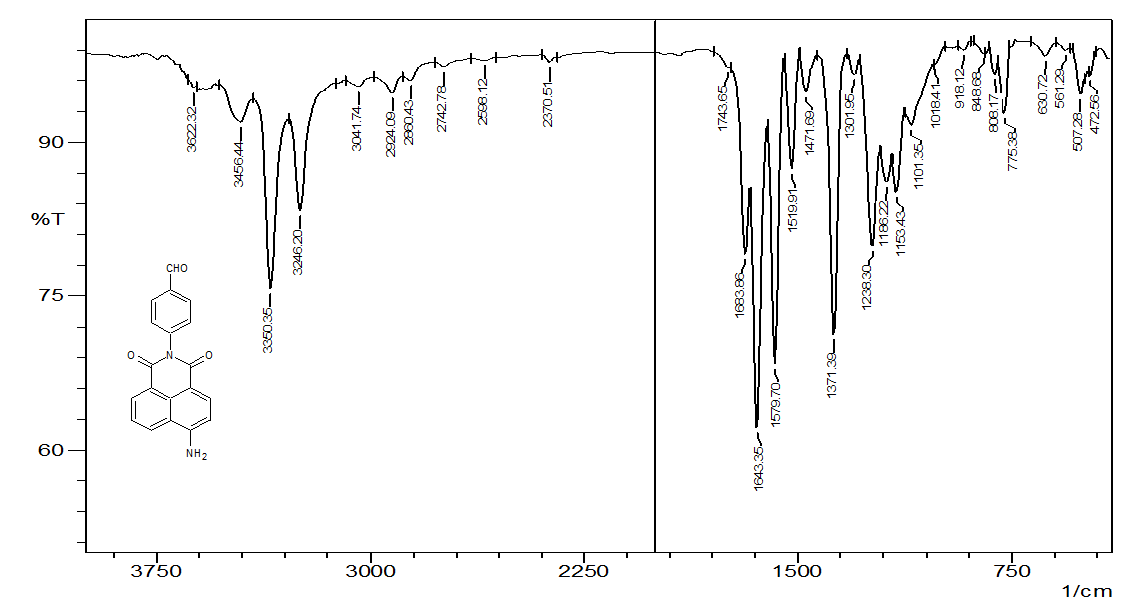


**Fig. S6** FT-IR spectrum of NIB-red


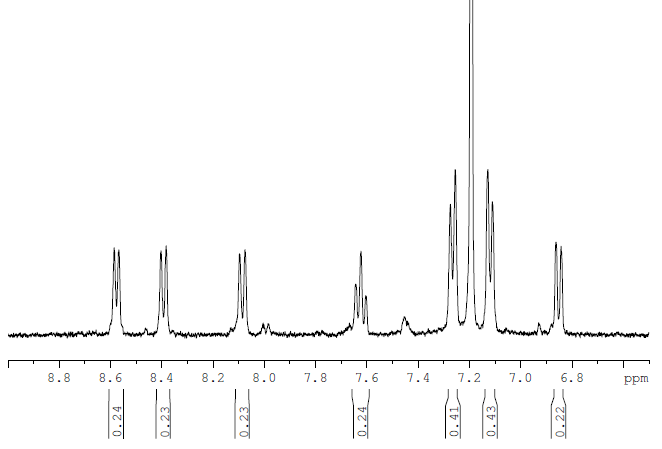

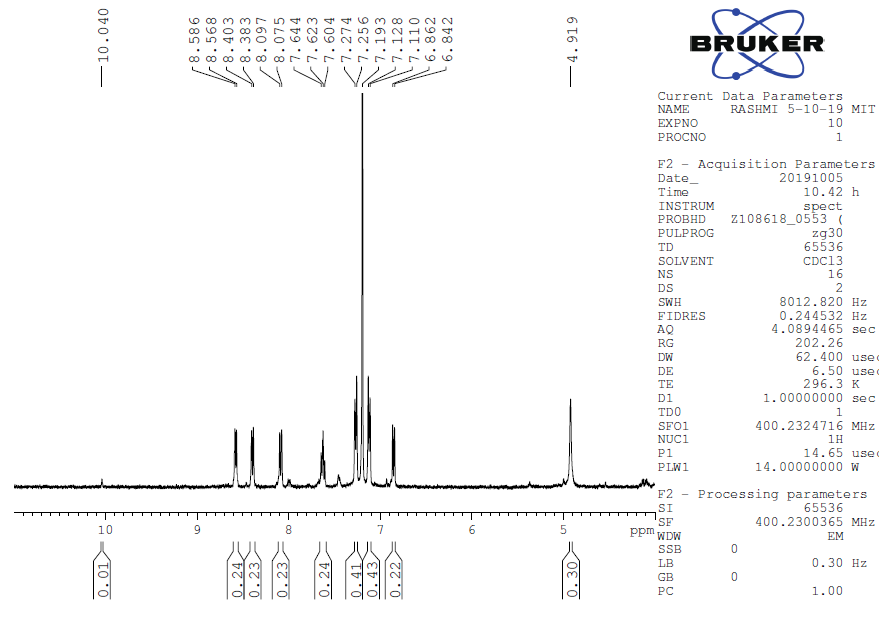


**Fig. S7** ^1^H NMR spectrum of NIB-red


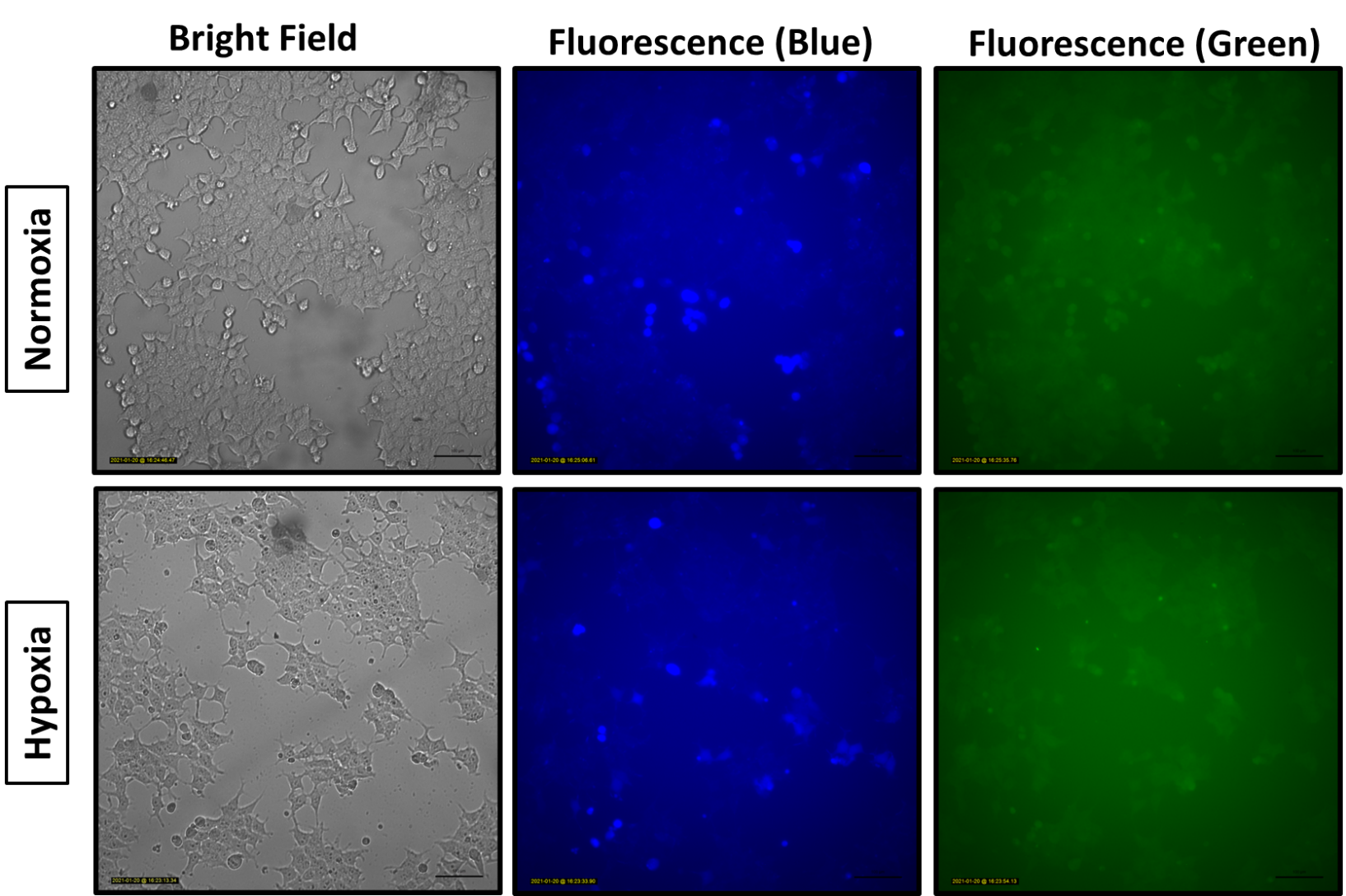


**Fig. S8** Fluorescence images of MCF-7 cells incubated with 6 µM NIB under normoxic (20% O_2_) and hypoxic (1 % O_2_) conditions, after 6 h of NIB incubation imaged before washing for bright and blue/green fluorescence (blue: excitation 370-410 nm; emission 429-462 nm; green: excitation 473-491 nm; emission 502-561 nm). Scale bar: 100 µm.


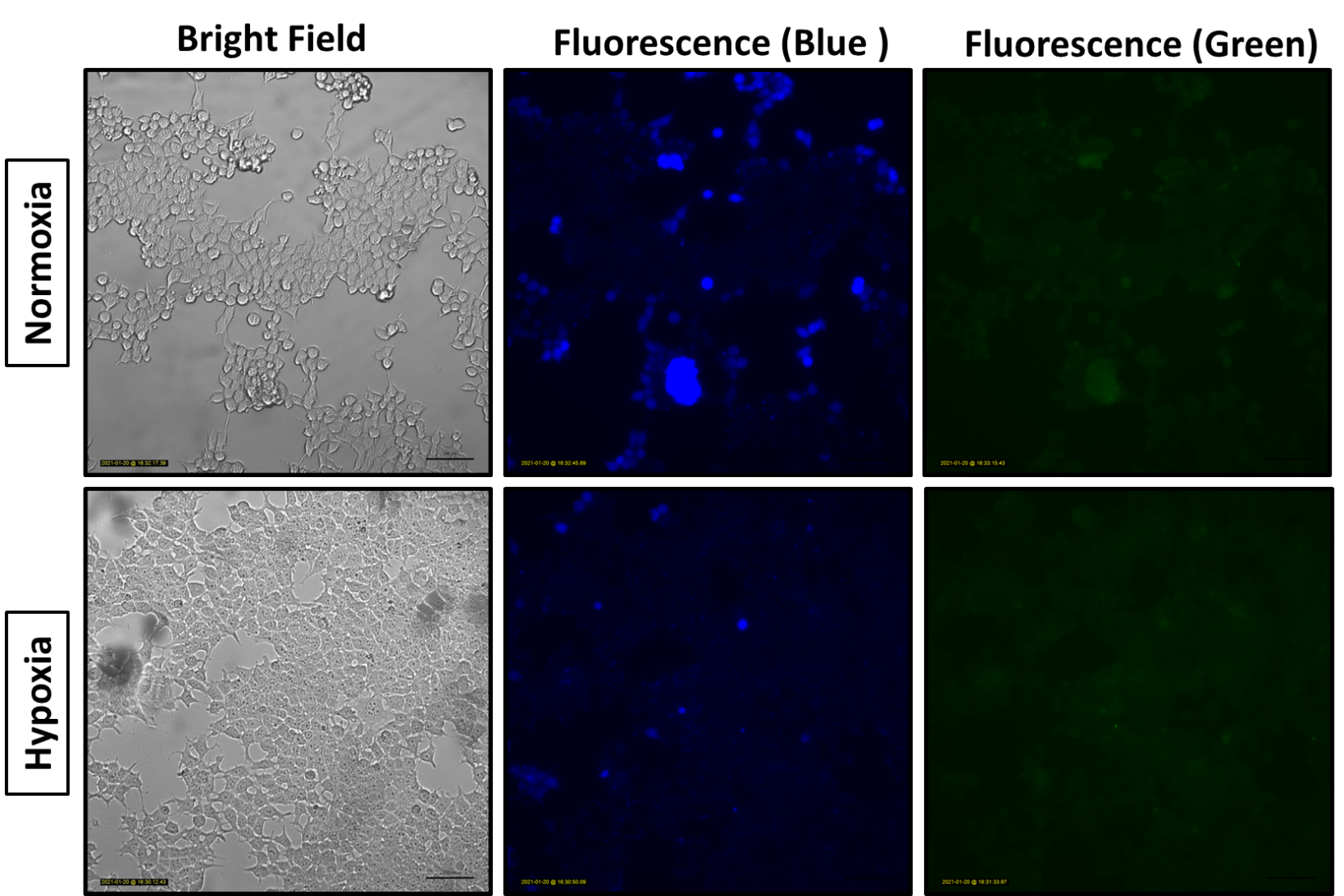


**Fig. S9** Fluorescence images of MCF-7 cells incubated with 6 µM NIB under normoxic (20% O_2_) and hypoxic (1% O_2_) conditions, after 6 h of NIB incubation imaged after washing for bright and blue/green fluorescence (blue: excitation 370-410 nm; emission 429-462 nm; green: excitation 473-491 nm; emission 502-561 nm). Scale bar: 100 µm.
